# RETRACTION: Twist1 Accelerates Tumour Vasculogenic Mimicry by Inhibiting Claudin15 Expression in Triple‐Negative Breast Cancer

**DOI:** 10.1111/jcmm.70162

**Published:** 2024-10-22

## Abstract

**RETRACTION**: D. Zhang, B. Sun, X. Zhao, H. Sun, J. An, X. Lin, D. Zhu, X. Zhao, X. Wang, F. Liu, Y. Zhang, J. Liu, Q. Gu, X. Dong, Z. Qiu, Z. Liu, H. Qi, N. Che, J. Li, R. Cheng and X. Zheng, “Twist1 Accelerates Tumour Vasculogenic Mimicry by Inhibiting Claudin15 Expression in Triple‐negative Breast Cancer,” *Journal of Cellular and Molecular Medicine* 24, no. 13 (2020): 7163–7174, https://doi.org/10.1111/jcmm.15167.

The above article, published online on 29 May 2020 in Wiley Online Library (wileyonlinelibrary.com), has been retracted by agreement between the authors; journal Editor‐in‐Chief, Stefan N. Constantinescu; the Foundation for Cellular and Molecular Medicine; and John Wiley & Sons Ltd. The retraction has been agreed as two images presented in figure 3b were found to be duplicated in figure 4b, but these images are described as representing different cells or different experimental conditions. Furthermore, a duplication of an image included in Figure 2 was found in a paper published earlier in another journal by some of the same authors. The authors provided some data and an explanation; however, this was not considered satisfactory. Given the extent of the identified issues, the editors have lost confidence in the data presented.

**RETRACTION**: D. Zhang, B. Sun, X. Zhao, H. Sun, J. An, X. Lin, D. Zhu, X. Zhao, X. Wang, F. Liu, Y. Zhang, J. Liu, Q. Gu, X. Dong, Z. Qiu, Z. Liu, H. Qi, N. Che, J. Li, R. Cheng and X. Zheng, “Twist1 Accelerates Tumour Vasculogenic Mimicry by Inhibiting Claudin15 Expression in Triple‐negative Breast Cancer,” *Journal of Cellular and Molecular Medicine* 24, no. 13 (2020): 7163–7174, https://doi.org/10.1111/jcmm.15167.

The above article, published online on 29 May 2020 in Wiley Online Library (wileyonlinelibrary.com), has been retracted by agreement between the authors; journal Editor‐in‐Chief, Stefan N. Constantinescu; the Foundation for Cellular and Molecular Medicine; and John Wiley & Sons Ltd. The retraction has been agreed as two images presented in figure 3b were found to be duplicated in figure 4b, but these images are described as representing different cells or different experimental conditions. Furthermore, a duplication of an image included in Figure 2 was found in a paper published earlier in another journal by some of the same authors. The authors provided some data and an explanation; however, this was not considered satisfactory. Given the extent of the identified issues, the editors have lost confidence in the data presented.

